# Dental implant nanotopography for peri-implant tissue stability: soft-tissue sealing, innate immune calibration, and active biointerface regulation

**DOI:** 10.1186/s40729-026-00703-4

**Published:** 2026-07-13

**Authors:** Takeru Kondo, Masahiro Yamada, Sara Ambo, Hiroshi Egusa

**Affiliations:** 1 Division of Molecular and Regenerative Prosthodontics, Tohoku University Graduate School of Dentistry, Sendai, Miyagi Japan; 2 Division of Next-Generation Dental Material Engineering, Tohoku University Graduate School of Dentistry, Sendai, Miyagi Japan; 3 Division of Mechanobiology and Biomedical-Dental Engineering, Tohoku University Graduate School of Biomedical Engineering, Sendai, Miyagi Japan; 4 Centre of Excellence for Dental Stem Cell Biology, Faculty of Dentistry, Chulalongkorn University, Pathumwan, Bangkok, Thailand; 5 Center for Advanced Stem Cell and Regenerative Research, Tohoku University Graduate School of Dentistry, Sendai, Miyagi Japan

**Keywords:** Dental implant, Peri-implantitis, Nanotopography, Innate immunity, Mechanotransduction, Soft-tissue sealing

## Abstract

**Purpose:**

Peri-implantitis is a major biological complication in implant dentistry, associated with bacterial biofilm formation and amplified by dysregulated host immune responses. Increasing evidence indicates that implant and abutment surface properties influence not only osseointegration but also microbial retention, soft-tissue sealing, and innate immune cell behavior. This narrative review aimed to summarize the current evidence on how dental implant nanotopography may affect peri-implantitis-related biological responses, with particular emphasis on innate immunity, region-specific peri-implant biointerfaces, and peri-implant tissue stability.

**Methods:**

Clinical, animal, and in vitro studies were reviewed to examine the pathogenesis of peri-implantitis, the roles of innate immune cells, the influence of implant and abutment surface topography on microbial and immune responses, and emerging nano-enabled strategies for the active regulation of peri-implant tissue responses.

**Results:**

Peri-implantitis progression is shaped by the interplay among the disruption of transmucosal soft-tissue sealing, microbial invasion, implant- or abutment-derived foreign-body stimuli, and sustained dysregulated innate immune activation. Nanotopographic surfaces regulate epithelial and fibroblastic attachment, extracellular matrix organization, neutrophil and macrophage responses, and osteocyte network formation via mechanotransduction. The biological effects of nanotopography are design-dependent and are influenced by nanoscale geometry, size, hydrophilicity, and associated physicochemical properties, including surface chemistry, wettability, and charge. Additionally, anisotropic nanospike surfaces and nano-enabled biomolecule delivery technologies illustrate the future possibility of active nanointerface regulation beyond conventional surface modification, although intracellular delivery technologies currently remain conceptual for dental implant applications.

**Conclusions:**

Implant and abutment nanotopography may contribute to peri-implant tissue stability by coordinating soft-tissue sealing, microbial retention control, innate immune calibration, and bone-interface resilience. Although direct clinical evidence remains limited, region-specific and functionally active nano-biointerfaces may offer new opportunities to reduce susceptibility to peri-implant inflammatory tissue breakdown.

## Introduction

Dental implants are widely used to rehabilitate edentulous areas, offering high success rates and predictable outcomes [1, 2]. However, the increasing prevalence of peri-implantitis, a chronic inflammatory condition characterized by peri-implant mucosal inflammation and progressive alveolar bone loss, poses a significant challenge to long-term implant success [3, 4]. The pathogenesis of peri-implantitis is multifactorial, involving bacterial biofilm formation on implant, abutment, and prosthetic surfaces, disruption of peri-implant soft-tissue sealing, implant-derived foreign body reactions, and dysregulated host immune responses [5, 6].

Although bacterial colonization is widely recognized as a major trigger of peri-implant inflammation, it has become increasingly clear that the host immune response, particularly the innate immune system, plays a decisive role in disease progression and tissue destruction [7]. Immune cells, including neutrophils, macrophages, and T and B lymphocytes, respond to microbial and implant- or abutment-derived stimuli by releasing excessive pro-inflammatory mediators and activating osteoclastogenesis, thereby contributing to peri-implant bone resorption [8, 9]. However, innate immune cells are also essential for microbial clearance, foreign-body surveillance, and wound healing. Therefore, peri-implant tissue stability requires appropriate calibration of host-defense and tissue-destructive immune responses rather than simple suppression of inflammation [7, 10].

Soft-tissue sealing in the transmucosal region represents the first biological barrier that separates the oral microbial environment from the underlying peri-implant tissues. Once this epithelial and connective tissue barrier is compromised, microbial ingress, bacterial corrosion of titanium surfaces, titanium ion or particle release, and persistent innate immune activation may mutually amplify peri-implant inflammation [11–14]. Accordingly, peri-implant soft-tissue sealing should be regarded not merely as a structural attachment, but as a physical–immunological barrier that regulates the local inflammatory microenvironment. Additionally, experimental evidence indicates that the mechanical properties and extracellular matrix integrity of gingival connective tissue can influence fibroblast-mediated inflammatory responses, thereby linking peri-implant mucosal stability to mechanobiological regulation [15, 16].

Parallel to advances in immunological understanding, considerable attention has been paid to the role of implant and abutment surface properties in peri-implant health [17]. Surface characteristics, such as roughness, chemistry, surface energy, and electrical properties, affect osseointegration, bacterial adhesion, epithelial and connective tissue attachment, immune cell behavior, and, in prosthetic components, interfacial performance such as cement bonding [10, 14, 18–22]. Recent studies have suggested that nanoengineered titanium surfaces can influence multiple events at the peri-implant biointerface, including anti-biofouling and mechano-bactericidal effects, macrophage phagocytosis, osteoclastogenesis-suppressive macrophage responses, and osteocyte network formation [19, 23–26]. These findings support the view that nanoengineered implant and abutment surfaces can function as active biointerfaces capable of coordinating microbial, immune, soft-tissue, and bone responses.

Importantly, these biological responses should not be interpreted as uniform “implant surface” effects, because the biological requirements differ substantially among peri-implant regions. Supramucosal and prosthetic surfaces require cleanability and biofilm control; the transmucosal abutment or implant neck requires epithelial attachment, fibroblast adhesion, collagen organization, and low bacterial retention; the implant–abutment junction is associated with microgap leakage, micromotion, wear debris, and corrosion; and the intraosseous implant fixture surface requires osseointegration, osteocyte network formation, and immune–bone coupling [10, 12–14, 17, 20–22, 25, 26]. Therefore, dental implant nanotopography should be discussed within a region-specific nano-biointerface framework rather than as a generalized surface property.

Mechanical stimuli and other physical cues from the surrounding microenvironment are sensed by cellular mechanosensors, converted into intracellular biochemical signals, and transmitted to the nucleus via cellular mechanotransduction to determine cell function and fate [27–31]. The topography and stiffness of the microenvironment provide static mechanical cues that regulate cellular functions, and biomaterial surfaces have been designed to control cell proliferation, differentiation, and immune responses through physical cues such as topography, stiffness, and spatial confinement [32–36]. Among these strategies, nanotopography on implant and abutment surfaces has attracted significant attention because of its beneficial effects on osseointegration and potential to regulate peri-implant tissue homeostasis [37–39]. Importantly, nanoscale structures on these surfaces may modulate immune responses by either attenuating excessive inflammation or enhancing host-defense functions, depending on their geometry, physicochemical properties, and biological context [23, 24, 36]. These findings suggest that nanotopographical features contribute to peri-implant tissue stability by regulating inflammatory reactions and tissue responses at region-specific implant and abutment interfaces.

In addition to providing cell-instructive physical cues, nanostructured surfaces may also promote biomolecular adsorption, conjugation, or spatial presentation, thereby creating future opportunities for local biomolecule delivery and immune-targeted surface functionalization [15, 33, 34, 40–48]. Although such nano-enabled delivery technologies remain largely experimental for dental implant applications, they provide a conceptual basis for future implant and abutment surfaces capable of actively modulating the region-specific peri-implant immune microenvironment.

Despite these insights, the relationship between implant and abutment surface nanotopography and peri-implantitis remains incompletely understood, particularly in terms of how nanotopographical cues interact with and coordinate soft-tissue sealing, microbial and foreign-body inflammatory triggers, innate immune regulation, and bone-interface resilience. This narrative review summarizes the current evidence regarding the effects of dental implant nanotopography on peri-implant tissue stability, with an emphasis on how nanotopographical cues regulate these interconnected biological processes. Because the available evidence includes both studies directly involving dental implant or abutment surfaces and studies using non-dental biomaterials or general mechanobiology models, their translational relevance to dental implant applications is considered according to the biological region and evidence level. As direct clinical evidence linking implant nanotopography to peri-implantitis prevention remains limited, mechanistic and translational studies are also discussed to clarify potential biological pathways and future implications for the design of next-generation implant and abutment surfaces that reduce susceptibility to peri-implant inflammatory tissue breakdown. In this review, peri-implant tissue stability is considered as an integrated biological state supported by four interrelated processes: soft-tissue sealing, microbial control, innate immune regulation, and bone-interface resilience. This framework provides a basis for understanding how implant nanotopography may contribute to peri-implant tissue homeostasis beyond osseointegration alone.

## Region-specific biological requirements of implant and abutment nano-biointerfaces

A key consideration in discussing implant and abutment surface properties is that peri-implant surfaces are not biologically uniform. The term “implant surface” is often used broadly to describe several anatomically and functionally distinct regions, including the supramucosal or prosthetic surface, the transmucosal abutment or implant neck, the implant–abutment junction, and the intraosseous implant fixture. Each region is exposed to different microbial, mechanical, immune, and tissue environments and therefore requires different surface-design priorities. Accordingly, implant and abutment surface properties should be interpreted within a region-specific biointerface framework rather than as a generalized surface property.

In supramucosal and prosthetic regions, cleanability, prosthetic contour, salivary pellicle formation, and biofilm control are critical [17, 20–22, 49]. In the transmucosal abutment or implant neck region, epithelial attachment, gingival fibroblast adhesion, collagen organization, extracellular matrix stability, and low bacterial retention are essential for establishing a stable soft-tissue seal [11, 14, 17, 50–55]. At the implant–abutment junction, microgap leakage, micromotion, wear debris, and corrosion may contribute to microbial accumulation and implant- or abutment-derived inflammatory stimuli [12, 13, 56, 57]. In contrast, the intraosseous implant fixture surface is primarily associated with osseointegration, bone remodeling, osteocyte network formation, and immune–bone coupling [10, 20, 25, 26, 58]. Thus, the biological value of a given surface feature depends strongly on its anatomical location and exposure status.

This region-specific framework provides the basis for interpreting the studies discussed in this review. Evidence derived from titanium implant or abutment surfaces is most directly relevant to dental implantology, whereas findings from non-dental biomaterials, nanowires, nanoneedles, nanostraws, or general mechanobiology models should be considered mechanistic or conceptual evidence. Throughout this review, we therefore distinguish, where appropriate, between direct implant-related evidence and extrapolated evidence, and discuss how nanotopographical cues may be translated into region-specific implant and abutment surface designs.

## Pathogenesis of peri-implantitis compared with periodontitis

Bacterial colonization of the peri-implant sulcular surface, abutment surface, prosthetic surface, or exposed implant surface is a major initiating factor in peri-implantitis, triggering inflammation within the peri-implant mucosa and subsequently contributing to progressive peri-implant bone loss [35, 59]. Clinically, peri-implantitis shares several features with periodontitis, including gingival inflammation, bleeding upon probing, and marginal radiographic bone loss. However, accumulating evidence indicates that the biological behavior and disease progression of peri-implantitis differ substantially from those of periodontitis [60]. Compared with periodontitis, peri-implantitis is characterized by a more rapid and aggressive disease course [61]. Histological analyses have demonstrated that inflammatory cell infiltration extends more apically in peri-implantitis lesions than in periodontitis, resulting in larger inflammatory lesions and accelerated marginal bone loss [62]. Consistent with these observations, peri-implant tissues exhibit increased infiltration of neutrophils, macrophages, and lymphocytes, accompanied by elevated expression of pro-inflammatory cytokines, including tumor necrosis factor (TNF)-α and interleukin (IL)-6, which are potent drivers of osteoclast activation and bone resorption [63]. These findings suggest that peri-implantitis is associated with a more extensive, osteolytic inflammatory response at the cellular level, which is distinct from that observed in periodontitis.

Traditionally, the absence of periodontal ligaments has been considered a key factor in the rapid progression of peri-implantitis [61]. Unlike natural teeth, implants lack a ligamentous barrier and vascular supply, potentially facilitating the spread of inflammation toward the alveolar bone. In addition to the absence of the periodontal ligament, fundamental structural differences in the peri-implant soft-tissue architecture further contribute to the distinct progression pattern of peri-implantitis compared with that of periodontitis [64]. Collagen fibers originating from the periodontal ligament are inserted perpendicularly into the cementum around natural teeth to form a firm connective tissue attachment. In contrast, the connective tissue surrounding the implants lacks periodontal ligament insertion, and collagen fibers are predominantly aligned parallel to the transmucosal implant or abutment surface [65]. Moreover, the peri-implant epithelium exhibits fewer hemidesmosomes and a less extensive basement membrane distribution than natural teeth, resulting in a weaker epithelial seal [11]. The connective tissue around the implants also contains fewer fibroblasts and blood vessels and demonstrates characteristics resembling scar-like tissue [66]. Collectively, these structural features weaken the peri-implant mucosal barrier, making it more susceptible to mechanical disruption, microbial penetration, and the apical spread of inflammatory infiltrates. Therefore, the distinct soft-tissue organization around implants not only compromises the stability of the biological seal but also reduces the ability of peri-implant tissues to compartmentalize microbial and inflammatory challenges, thereby explaining the accelerated inflammatory spread and aggressive tissue breakdown characteristics of peri-implantitis [14].

Recent studies have highlighted implant-associated foreign body reactions as another important pathogenic mechanism in peri-implantitis [6]. Titanium implants are known to release titanium ions and particles into surrounding tissues during placement, functional loading, and micromotion at the bone–implant interface [12, 57]. In addition, implant- or abutment-derived debris and corrosion products may arise from several region-specific sources, including implant placement, micromotion at the bone–implant interface, wear or fretting at the implant–abutment junction, mechanical instrumentation, and biofilm-mediated corrosion on exposed titanium surfaces [12, 13, 56, 57]. Importantly, oral bacterial biofilms further exacerbate this process by inducing corrosion and degradation of the titanium oxide layer. Culturing oral bacteria such as *Streptococcus sanguinis* and *Porphyromonas gingivalis* on titanium surfaces significantly increases titanium ion release [13]. Titanium particles around implants have been shown to elevate the expression of inflammatory mediators including IL-6, TNF-α, and receptor activator of nuclear factor-κB ligand [67]. These findings suggest that titanium-derived ions and particles amplify inflammatory responses at the peri-implant mucosal tissue and bone–implant interfaces. Consequently, bacterial dysbiosis and implant-derived foreign body reactions may create an implant-specific inflammatory microenvironment that accelerates peri-implant tissue breakdown. Importantly, these pathogenic stimuli should not be attributed to a single generalized implant surface, because bacterial accumulation, microgap leakage, corrosion, wear debris, and bone-interface micromotion may occur at different anatomical regions and may contribute differently to peri-implant inflammation. Collectively, these structural and implant-associated factors provide a biological basis for sustained and dysregulated innate immune activation in peri-implantitis.

## Innate immunity in peri-implantitis

In peri-implantitis, microbial invasion, disruption of soft-tissue sealing, and region-specific implant- or abutment-derived foreign body stimuli converge to activate innate and adaptive immune responses [7] (Table 1). Among these cells, neutrophils and macrophages play central roles in establishing the pro-inflammatory and osteolytic microenvironments.

**Table 1:** Summary of studies examining the role of immune-cell involvement in peri-implantitis

Cell type	Study type	Key findings	References
Neutrophils	Experimental study	*P. gingivalis* lipopolysaccharide promoted peri-implant inflammation through neutrophil activation	Wang et al. [72]
	Human study	CXCR2 expressing neutrophils increased in peri-implantitis tissue	Mo et al. [70]
		Elevated CXCL5 and CXCL8 expression was associated with neutrophil infiltration in peri-implantitis	Park et al. [71]
		Neutrophils were recruited by CXCL13 + fibroblasts through CXCL8/CXCL6-CXCR2/CXCR1	Li et al. [8]
Macrophages	Human study	Peri-implantitis tissue showed abundant M1 macrophages compared with M2 macrophages	Galarraga-Vinueza et al. [77]
		A higher number of M1 macrophages was observed in peri-implantitis tissue than M2 macrophages	Dionigi et al. [78]
		The proportion of M1 macrophages was higher in peri-implantitis tissue than in periodontitis tissue	Fretwurst et al. [79]
		Peri-implantitis tissues showed upregulation of M2 macrophage–associated and tissue remodeling–related gene signatures	Oh et al. [60]
	Animal study	Macrophage depletion in a murine peri-implantitis model reduced inflammatory responses and peri-implant bone loss	Ginesin et al. [73]
		CCR2 knockout mice showed decreased macrophage infiltration, leading to reduced inflammation and bone loss around the implants	Yuan et al. [80]
		Inhibition of pro-inflammatory macrophage responses suppressed bone resorption	Zhao et al. [81]
Regulatory T cells	Human study	Peri-implantitis lesions exhibited increased infiltration of regulatory T cells but impaired regulatory function	Cafferata et al. [86]
B cells	Human study	Peri-implantitis tissue showed an increased frequency of B cells compared with healthy gingiva	Malmqvist et al. [87]
NK cells and macrophages	Human study	Greater number of NK cells and macrophages were detected in peri-implantitis tissue than the healthy counterpart tissue	Villalobos et al. [88]

Neutrophils are among the first responders to microbial invasion and are abundantly present in peri-implantitis tissues [68]. Their activation leads to the release of pro-inflammatory cytokines, including IL-1β and TNF-α, which in turn amplify local inflammation and promote osteolytic activity in peri-implantitis [69]. Recent single-cell and clinical studies have shown that neutrophil recruitment in peri-implantitis is associated with C-X-C motif chemokine receptor (CXCR)2-expressing neutrophils, elevated C-X-C motif chemokine ligand (CXCL)5/CXCL8 levels, and fibroblast-derived chemokine axes involving CXCL8/CXCL6–CXCR2/CXCR1 [8, 70, 71]. These findings indicate that neutrophils are actively recruited through chemokine-mediated cell–cell interactions and contribute to inflammatory amplification and bone resorption. More recently, *Porphyromonas gingivalis* lipopolysaccharide was shown to promote peri-implant inflammation through neutrophil activation, further supporting neutrophils as key effector cells linking bacterial virulence factors to inflammatory tissue breakdown around implants [72].

Macrophages play key roles in peri-implantitis progression [73]. These cells exhibit polarization plasticity: M1 macrophages produce pro-inflammatory cytokines such as IL-1β, IL-6, and TNF-α, whereas M2 macrophages release anti-inflammatory cytokines, including IL-4 and IL-13 [74–76]. Human peri-implantitis tissues are enriched with M1-like macrophages, and a higher proportion of M1 macrophages has been reported in peri-implantitis tissues than in periodontitis tissues [77–79]. Animal studies further support the pathogenic role of macrophages, as macrophage depletion, C–C motif chemokine receptor 2 deficiency, or inhibition of pro-inflammatory macrophage responses reduces peri-implant inflammation and bone resorption [73, 80, 81]. However, transcriptomic analyses of human peri-implantitis tissues have also reported the upregulation of M2 macrophage–associated and tissue remodeling–related gene signatures, suggesting mixed macrophage activation rather than a simple M1/M2 dichotomy [60]. Thus, although the precise contribution of macrophage polarization to the pathogenesis of peri-implantitis remains unclear, macrophage-derived inflammatory and tissue-remodeling mediators clearly contribute to disease initiation, persistence, and progression.

In addition to their accumulation in response to bacterial invasion, macrophages are central mediators of the foreign body reaction and serve as precursors to multinucleated giant cells, which are a defining feature of this response [82]. Multinucleated giant cells arise through macrophage fusion in response to persistent implant-derived stimuli, such as titanium ions and particles, and are frequently observed at the bone–implant interface [83]. Their presence in peri-implant tissues reflects sustained macrophage activation and may contribute to the chronic inflammation associated with implant-derived foreign body reactions.

Lymphocytes, including T and B cells, have also been implicated in the progression of peri-implantitis. T helper 17 cells secrete IL-17, which promotes neutrophil recruitment and enhances osteoclastogenesis, thereby exacerbating peri-implant bone resorption [84, 85]. Human peri-implantitis lesions exhibit increased infiltration of regulatory T cells, which normally suppress excessive immune responses; however, their regulatory function appears to be impaired, suggesting that functionally compromised regulatory T cell-mediated immune regulation contributes to persistent inflammation in peri-implantitis [86]. Increased frequencies of B and natural killer cells have also been reported in peri-implantitis tissues compared with their healthy counterparts [87, 88]. Recent research has highlighted the role of group 3 innate lymphoid cells (ILC3s) in periodontitis, which contribute to tissue inflammation and bone resorption [89]. Although direct evidence of ILC3 involvement in peri-implantitis is currently lacking, its established role in periodontal disease suggests that ILC3s could similarly participate in the pathogenesis of peri-implantitis, representing a potential area for future investigation.

**Fig. 1:**
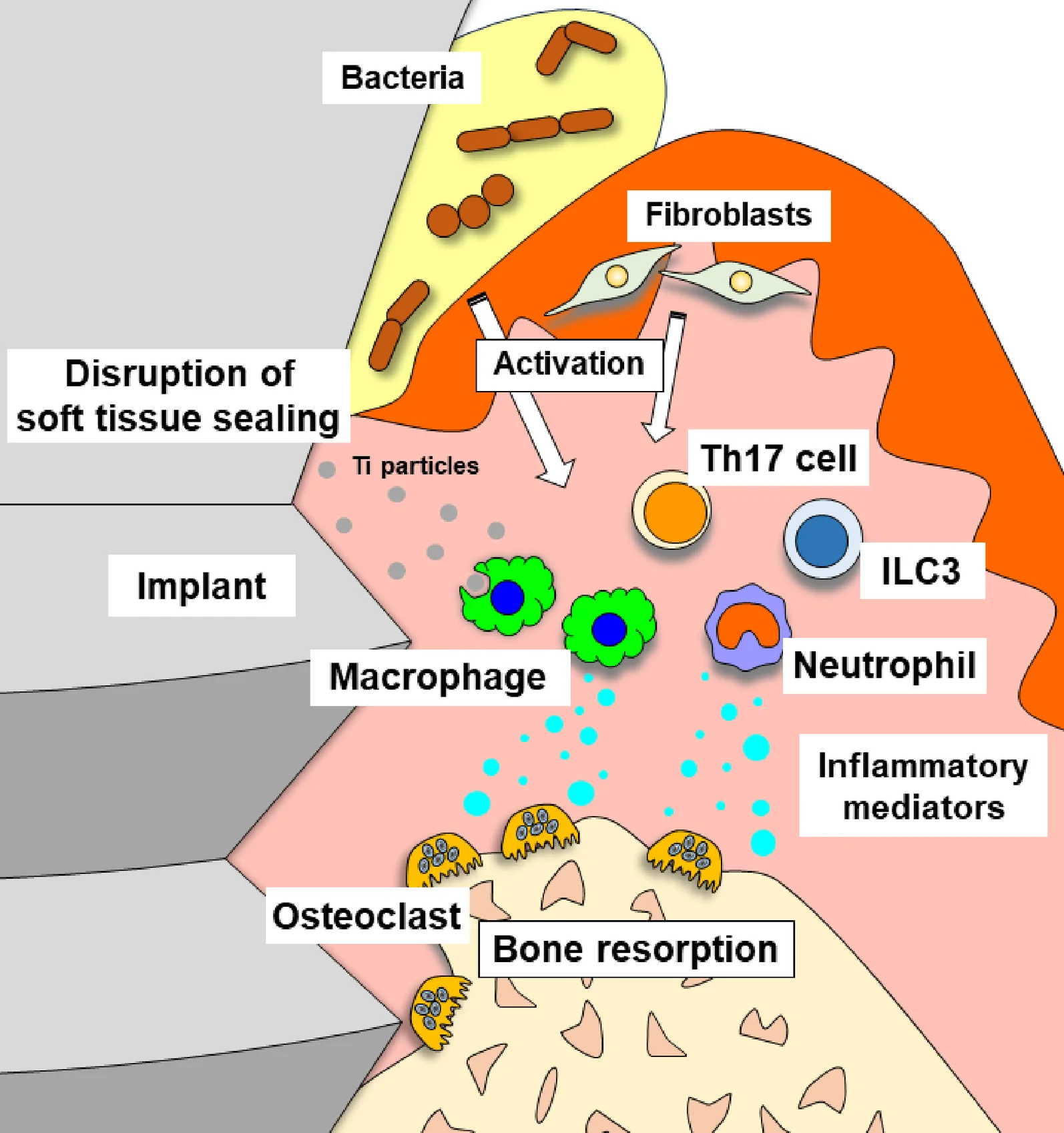
Molecular and cellular mechanisms involved in the initiation and progression of peri-implantitis. Bacterial accumulation at the peri-implant sulcus disrupts soft-tissue sealing and allows microbial infiltration into the peri-implant mucosa. Microbial challenge and implant-derived titanium particles activate gingival fibroblasts and immune cells, including macrophages, neutrophils, and T helper 17 (Th17) cells, thereby releasing inflammatory mediators. These inflammatory mediators promote osteoclastogenesis and osteoclast-mediated bone resorption, thereby driving progressive peri-implant tissue breakdown. Group 3 innate lymphoid cells (ILC3s) are identified as potential contributors given their established role in periodontal inflammation

Collectively, microbial infection, disruption of soft-tissue sealing, and implant-derived foreign body reactions induce sustained activation of innate immune cells, disrupting the balance between osteoclast and osteoblast activities within peri-implant tissues [90]. Osteocytes also contribute to bone loss by secreting signaling molecules that regulate bone remodeling, thereby further contributing to the pathogenesis of peri-implantitis [58]. Peri-implantitis is characterized by sustained and dysregulated immune activation that creates a pro-inflammatory and osteolytic microenvironment, ultimately driving peri-implant bone resorption (Fig. 1). These immune responses should be interpreted in relation to the regional source of stimulation, because microbial challenge at the peri-implant sulcus, disruption of the transmucosal soft-tissue seal, implant–abutment junction-related stimuli, and bone–implant interface reactions may each contribute to peri-implant inflammation through distinct but interconnected pathways.

## Soft-tissue sealing in peri-implantitis

Soft-tissue sealing in the transmucosal region of dental implants represents a critical biological barrier that separates the oral microbial environment from the underlying peri-implant tissues [17]. Insufficient soft-tissue sealing allows pathogens to penetrate the peri-implant mucosa. Therefore, an intact epithelial and connective tissue seal functions as a physical–immunological barrier that limits microbial ingress, thereby reducing the risk of biofilm-associated titanium corrosion, titanium ion or particle release, and the subsequent innate immune activation. Thus, the disruption of soft-tissue sealing can be regarded as an upstream event that links the microbial challenge to persistent peri-implant inflammation and bone resorption [91].

The quality of peri-implant soft-tissue sealing depends not only on epithelial and fibroblastic attachment to the transmucosal surface but also on the integrity, organization, and mechanical properties of the surrounding extracellular matrix. Evidence from gingival and periodontal tissue models indicates that matrix stiffness and collagen integrity regulate both stromal and immune responses. In gingival fibroblasts, substrate stiffness regulates inflammatory responses and extracellular matrix-related gene expression, suggesting that the mechanical microenvironment contributes to connective tissue stability and fibroblast-mediated inflammation [16, 17, 92]. Gingival extracellular matrix softening and collagen disorganization have been shown to enhance fibroblast-mediated inflammatory responses, whereas stiffness resembling healthy gingiva suppresses Toll-like receptor-mediated cytokine production and supports gingival immune homeostasis [93]. Extracellular matrix (ECM) mechanics also influence macrophage behavior; substrate stiffness modulates macrophage polarization, phagocytic capacity, and migration mode [94], whereas Piezo1-dependent Ca^2^⁺ influx mediates macrophage stiffness sensing and inflammatory activation on stiff substrates [95]. Moreover, macrophage Piezo1 signaling has been implicated in matrix metalloproteinase-mediated collagen degradation and gingival destruction in periodontitis [96], and collagen deposition/organization further modifies stiffness-dependent macrophage inflammatory responses [97]. Taken together, these findings suggest that soft-tissue sealing should be understood not only as a physical attachment to the transmucosal implant or abutment surface but also as a mechanobiologically regulated barrier that integrates fibroblast–ECM–macrophage crosstalk within the local immune microenvironment. Although much of this evidence is derived from gingival, periodontal, or biomaterial models rather than direct peri-implantitis models, it provides a mechanistic basis for considering peri-implant soft-tissue sealing as an immunoregulatory interface.

From a surface design perspective, nanotopography and physicochemical surface modification are particularly relevant to the transmucosal region because they regulates gingival fibroblast behavior and ECM organization. Beyond nano-ordered spikes and pores, several nanoengineered or surface-energy-enhanced titanium-based surfaces, including ultraviolet (UV)- or vacuum UV (VUV)-photofunctionalized titanium, titanium dioxide nanotubes, nanopores, nanocavities, highly ordered nanotube-like structures, and micro-/nano-structured tantalum coatings, have been reported to improve human gingival fibroblast attachment, proliferation, migration, and adhesion-related gene expression, supporting the broader concept that nanoscale and physicochemical surface designs can promote transmucosal soft-tissue integration [21, 50, 51, 55, 98]. Nano-textured and UV-photofunctionalized titanium surfaces promote the proliferation, spreading, attachment, adhesion, and retention of epithelial cells compared with unmodified titanium surfaces [54, 99]. Nanotubes on titanium surfaces facilitate the adhesion of gingival fibroblasts and epithelial cells compared with polished titanium, significantly enhancing soft-tissue sealing in vivo [52]. A titanium surface with nano-ordered spikes and pores created by alkali heat treatment enhances gingival fibroblast spreading, collagen synthesis, and collagen fiber organization, resulting in periodontal-like connective tissue attachment to titanium [53]. Although this finding does not directly demonstrate prevention of peri-implantitis, it supports the concept that a biomimetic nanosurface design may reinforce peri-implant soft-tissue sealing by promoting connective tissue integration at the transmucosal interface. Accordingly, in this region, nanotopography should be evaluated primarily as a transmucosal implant or abutment surface feature that may support epithelial and connective tissue integration, rather than as a generalized implant surface property.

## Soft-tissue integration versus microbial retention at the transmucosal interface

A central challenge in transmucosal surface design is the trade-off between soft-tissue integration and microbial retention. Surface features that promote host-cell adhesion, spreading, and extracellular matrix organization do not necessarily suppress bacterial adhesion. In the oral environment, implant and abutment surfaces are rapidly conditioned by salivary pellicles and adsorbed proteins, and surface topography, chemistry, wettability, and charge may influence not only the amount of protein adsorption but also the composition, conformation, orientation, and spatial distribution of the adsorbed protein layer [18, 35, 59, 100–102]. Because this conditioned protein layer can become the actual interface recognized by bacteria, the effect of nanotopography on microbial retention should be interpreted as a combined effect of direct surface–bacterial interactions and indirect protein-mediated interactions. Therefore, the concept that nanoscale features may promote epithelial or fibroblastic attachment without increasing bacterial retention should be interpreted cautiously and in a design-dependent manner.

This distinction is particularly important because nanoscale features and large micro-retentive roughness are not equivalent. Nanoscale architectures may provide host-cell-instructive physical cues without necessarily creating large protected niches for bacterial entrapment, whereas rough implant surfaces that are beneficial for osseointegration may become problematic if exposed to the oral cavity after crestal bone loss because of increased plaque accumulation and biofilm retention [19, 35, 59, 103]. Thus, the biological value of surface roughness or nanotopography depends on the anatomical region and exposure status. In the transmucosal abutment or implant neck region, the desired design principle is not simply to maximize cell attachment, but to promote epithelial and connective tissue sealing while minimizing microbial retention.

**Fig. 2:**
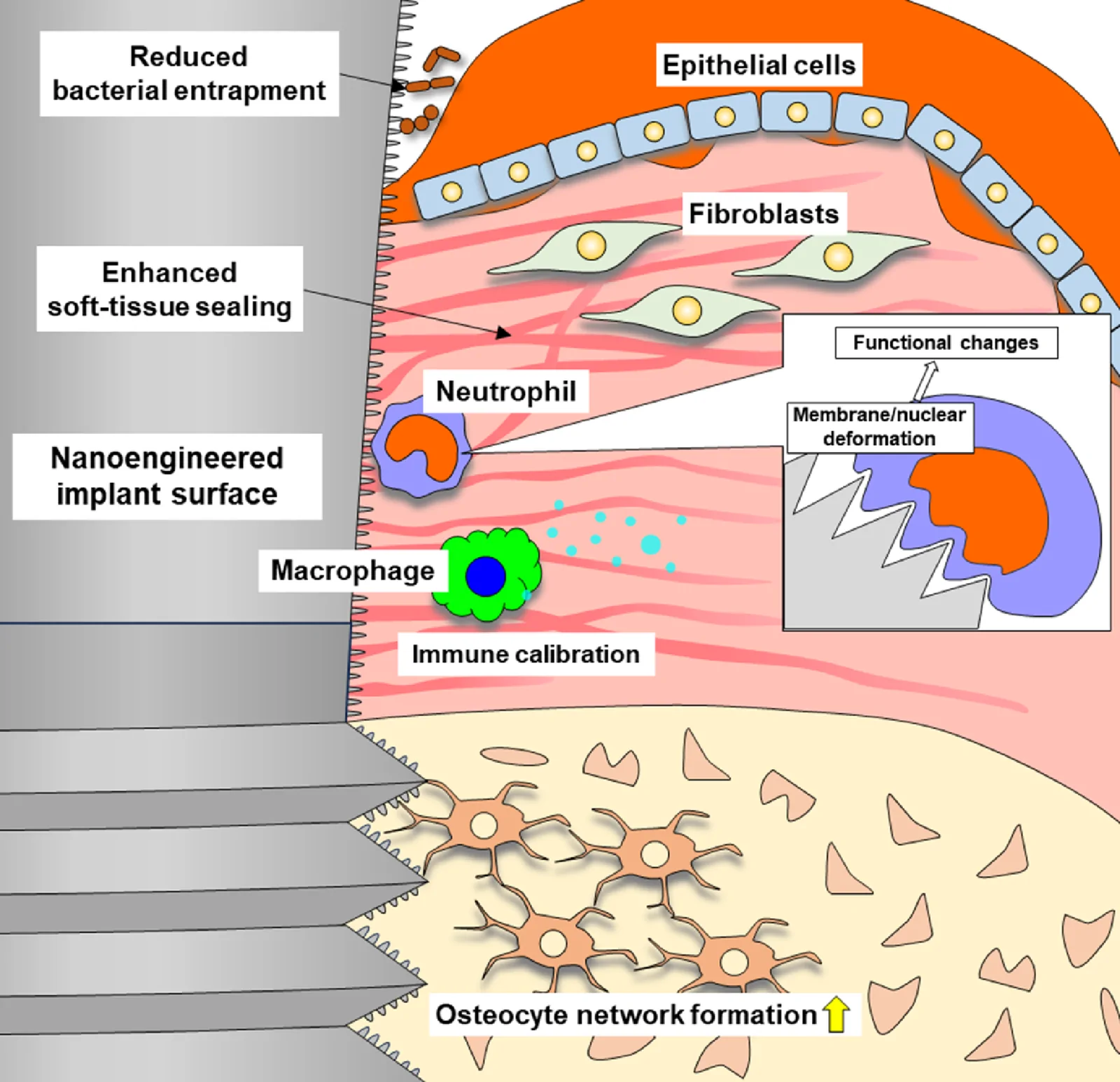
Proposed mechanisms by which nanoengineered implant surfaces support peri-implant tissue stability. Nanotopography at the transmucosal implant surface may enhance soft-tissue sealing by promoting epithelial attachment, gingival fibroblast responses, and collagen fiber organization at the implant–mucosa interface. Nanoscale surface features may also reduce bacterial entrapment at the peri-implant sulcus while maintaining host-cell-instructive cues. At the soft-tissue interface, nanotopography can modulate neutrophil and macrophage inflammatory responses through mechanotransductive processes involving membrane and nuclear deformation, leading to functional changes in immune cells. Nanotopography may promote osteocyte network formation at the bone interface, thereby contributing to its bone-interface resilience. Taken together, these coordinated effects may help reduce susceptibility to peri-implant inflammatory tissue breakdown. In some designs, the nanoscale architecture may also generate active physicochemical cues, such as electrical or mechano-bactericidal effects

In combination with enhanced hydrophilicity, altered surface charge, and, in some designs, mechano-bactericidal activity, nanosurfaces may help balance soft-tissue integration with reduced microbial retention at the transmucosal interface [19, 20]. Taken together, these findings support the concept that nanoengineered transmucosal implant or abutment surfaces may reinforce soft-tissue sealing while limiting bacterial entrapment at the implant–mucosa interface, thereby contributing to the coordinated regulation of microbial, immune, and bone-interface responses (Fig. 2). Accordingly, soft-tissue sealing may be viewed as an upstream contributor to peri-implant tissue stability, as it helps regulate subsequent microbial ingress, innate immune activation, and inflammatory tissue breakdown.

## Implant and abutment surface topography as a regulator of peri-implant microbial and immune responses

The surface properties of implant and abutment surfaces play a crucial role not only in osseointegration but also in microbial colonization, soft-tissue integration, and host immune responses at region-specific peri-implant biointerfaces [49]. Among these properties, implant surface roughness and topographical architecture have been widely studied because they can influence both bacterial retention and peri-implant tissue response.

Clinical evidence on the relationship between implant surface roughness and peri-implantitis remains controversial. Some systematic reviews have suggested that machined surfaces may show slightly more favorable outcomes in peri-implant marginal bone loss or probing pocket depth, raising concerns that rougher surfaces could promote plaque accumulation and bacterial colonization [103]. Conversely, other clinical studies and long-term observations have reported that rough-surfaced implants do not necessarily increase peri-implantitis-related outcomes and may contribute to more stable marginal bone levels or lower implant failure rates, possibly reflecting their established benefits for osseointegration and bone-interface stability [104–106]. Therefore, the current evidence does not support the simple conclusion that either smooth or rough surfaces are uniformly protective against peri-implantitis. Rather, the biological significance of surface roughness should be interpreted according to anatomical location and exposure status: roughness may be advantageous at the bone–implant interface for osseointegration, whereas exposed rough implant threads after crestal bone loss may increase plaque retention and complicate biofilm control.

Importantly, the biological impact of the implant surface topography cannot be explained solely by bacterial retention. Surface topography also directly influences host immune responses at the implant–tissue and abutment–tissue interfaces [10]. Surface features, such as roughness, ridges, grooves, and patterning, can modulate the behavior of innate immune cells, including neutrophils and macrophages. For example, microrough surfaces have been reported to attenuate pro-inflammatory neutrophil responses and promote anti-inflammatory cytokine production by macrophages [10], whereas patterned ridges or grooves can guide neutrophil migration better than smooth surfaces [107]. These findings indicate that surface topography can function as an active physical cue that shapes the local immune microenvironment, depending on the region in which it is applied.

Taken together, the implant and abutment surface design should be considered as a balance among osseointegration, microbial retention, soft-tissue sealing, and immune regulation rather than as a simple smooth-versus-rough comparison. This distinction is particularly important when considering nanotopography, because nanoscale surface features may provide cell-instructive cues without necessarily forming large micro-retentive niches that favor bacterial entrapment. Region-specific implant and abutment nanotopography represents a promising surface design strategy to integrate soft-tissue sealing, microbial retention control, innate immune modulation, and bone-interface resilience (Fig. 2).

## Nanotopography-mediated mechanotransduction and design-dependent immune calibration

The nanotopographical features of implant and abutment surfaces provide physical cues that can be sensed by immune cells through mechanotransduction, thereby influencing cell morphology, activation, and function. Evidence supporting this concept includes both titanium-based implant-relevant studies and mechanistic studies using non-dental nanostructured platforms; therefore, the translational relevance of each study should be interpreted according to the material, cell type, and experimental context. Macrophages cultured on nanorods with radii of 200 nm or larger exhibit elongated morphology, suggesting the activation of mechanotransductive pathways [108]. Mouse natural killer cells cultured on zinc oxide nanowires showed enhanced ligand–receptor interactions and cell contraction due to nanoscale cell-surface deformation, leading to increased activation [109]. In addition to membrane deformation, nanotopography can induce nuclear deformation through actin cytoskeletal forces, particularly around nanopillars [110]. Such membrane, cytoskeletal, and nuclear responses depend on the cell type and nuclear stiffness and are increasingly recognized as key mechanisms by which topographical cues regulate cellular behavior [111, 112]. Thus, nanotopography should be viewed as a physical regulator of immune cell mechanobiology and not merely a passive surface feature.

Representative studies have indicated that nanotopography can attenuate excessive inflammatory responses in innate immune cells across several experimental settings (Table 2). Titanium-based nanostructures provide relatively direct implant-relevant evidence. Titanium nanotube surfaces suppressed adhesion, proliferation, and pro-inflammatory cytokine production, including IL-6 and TNF-α, in neutrophils and macrophages compared with smooth titanium surfaces [113]. Titanium dioxide nanotubes also inhibited IL-6 and TNF-α production in RAW 264.7 macrophages [114]. Similarly, nanorough titanium surfaces restricted macrophage motility and reduced inflammatory cytokine expression compared with flat titanium surfaces [115], while anodized titanium–zirconia nanochannels inhibited TNF-α production in macrophages [116]. In contrast, findings from non-titanium nanoscale materials should be regarded as mechanistic or conceptual evidence for immune regulation rather than direct evidence for dental implant surfaces. Other nanoscale materials, including nanocrystalline hydroxyapatite, silicon-substituted hydroxyapatite, and nanocrystalline calcium phosphate, have also been reported to shift macrophage responses toward less inflammatory phenotypes or reduce the expression of pro-inflammatory mediators [117, 118]. These findings support the concept that nanoscale surface features can dampen excessive inflammatory activation, although their effects are highly dependent on material composition, geometry, and cellular context.

**Table 2:** Summary of studies investigating the effects of nanotopography on immune cell function and their evidence relevance to dental implant surfaces

Evidence relevance	Material	Nanoscale feature	Cell type	Key findings	References
Titanium-based implant-relevant mechanistic evidence	Titanium		Neutrophil; macrophage	Titania nanotube arrays reduced adherent immune-cell numbers and suppressed proliferation and pro-inflammatory cytokine production, including IL-6 and TNF	Smith et al. [113]
	Titanium dioxide		Macrophage	Titania nanotube-modified titanium reduced IL-6 and TNF-α production in RAW 264.7 macrophages	Neacsu et al. [114]
	Titanium		Macrophage	Nanostructured titanium restricted macrophage movement and suppressed TNF-α production	Lee et al. [115]
	Titanium zirconia alloy		Macrophage	Nanochannels formed on TiZr alloy suppressed TNF-α production in macrophages	Ion et al. [116]
	Titanium		Macrophage	Titanium nanospikes enhanced macrophage host-defense–related functions, including TNF-α expression and phagocytosis, whereas conditioned medium from nanospike-stimulated macrophages inhibited osteoclastogenesis	Kartikasari et al. [23, 24]
Non-titanium biomaterial mechanistic evidence	Nanocrystalline hydroxyapatite		Macrophage	Nanocrystalline hydroxyapatite promoted macrophage polarization from an M1-like toward an M2-like phenotype	Linares et al. [117]
	Nanocrystalline calcium phosphate		Macrophage	Nanoscale calcium phosphate modification suppressed the expression of inflammatory genes, including TNF-α, IL-6, CCL2, and CXCL12	Hamlet et al. [118]
	Platinum-based bulk metallic-glass alloy		Macrophage	Macrophage morphology and cell size changed depending on nanorod radius	Padmanabhan et al. [108]
	Zinc oxide		Natural killer cell	Nanoscale cell-surface deformation enhanced ligand–receptor interactions, cell contraction, and activation	Le Saux et al. [109]

However, nanotopography does not simply suppress inflammation. Certain nanostructures may induce functional immune activation that supports host defense while limiting tissue-destructive responses. Titanium nanospikes enhanced macrophage inflammatory activity, including TNF-α production, but conditioned media from macrophages cultured on nanospikes inhibited osteoclastogenesis [23]. Titania nanospikes also activated macrophage phagocytosis via ligand-independent contact stimulation [24]. These findings suggest that nanospikes may calibrate macrophage function by enhancing phagocytic competence and host-defense activity while generating signals that suppress osteoclast differentiation. This dual effect is particularly important in the context of peri-implantitis, where immune modulation should preserve microbial clearance while preventing excessive osteoclastogenic inflammation. Thus, nanotopography-mediated immune regulation should be understood as a functional immune calibration rather than simple anti-inflammatory suppression.

Immune responses to nanotopography are highly dependent on surface design parameters such as hydrophilicity, nanoscale size, and geometric organization (Table 3). Large-diameter hydrophilic titanium nanotubes promoted M1 macrophage polarization with increased pro-inflammatory cytokine expression through focal adhesion kinase–mitogen-activated protein kinase signaling [119], whereas small-diameter hydrophilic nanotubes preferentially induced M2-like polarization of human monocytes and promoted the osteogenic activity of bone marrow-derived stromal cells through macrophage-conditioned media [120, 121]. Geometric orientation also influences macrophage phenotype: aligned nanofiber surfaces skewed macrophages toward an M2-like phenotype and suppressed pro-inflammatory mediator production, whereas randomly oriented nanofibers promoted M1-associated cytokine secretion [122, 123]. These findings indicate that nanoscale immune regulation is design-dependent and that the biological outcome of nanotopography varies according to the surface size, hydrophilicity, orientation, and cellular context. Therefore, nanoengineered implant surfaces should be optimized according to the intended biological function at each implant region, rather than simply designed to increase or decrease inflammation.

**Table 3:** Summary of studies investigating the effects of hydrophilicity and geometrical organization of nanotopography on macrophage responses

Evidence relevance	Surface properties	Material	Biological model	Key conclusions	References
Titanium-based implant-relevant mechanistic evidence	Hydrophilicity/nanotube diameter	Titanium	In vivo	Large-diameter hydrophilic nanotubes promoted M1-like macrophage polarization	He et al. [119]
			In vitro	Hydrophilic titanium nanotubes regulated macrophage responses in a diameter-dependent manner, with large-diameter nanotubes promoting M1-like polarization and small-diameter nanotubes inducing M2-like, pro-osteogenic macrophage-mediated effects	Ma et al. [120] Wang et al. [121]
Non-titanium biomaterial mechanistic evidence	Geometrical organization	Nanofiber scaffold	In vitro	Aligned nanofiber surfaces promoted M2-like macrophage polarization and suppressed TNF and IL-6 production, whereas randomly oriented nanofiber surfaces induced M1-associated marker expression	Jia et al. [122] Schoenenberger et al. [123]

An important interpretative limitation is that biological responses cannot always be attributed to nanotopography alone. Nanotopographic processing often changes oxide chemistry, hydrophilicity, surface charge, carbon contamination, elemental composition, and other physicochemical cues [19, 23–25, 36, 113–121]. Unless nanoscale geometry is experimentally isolated from these variables, the observed effects should be interpreted as responses to nanotopography-associated physicochemical properties. Future studies should decouple geometry, chemistry, wettability, charge, and contamination to clarify which surface parameters are responsible for immune calibration at implant and abutment biointerfaces.

In addition to direct immune cell regulation, nanotopography may support peri-implant tissue stability by reinforcing the bone interface. Titanium implants with nanospikes enhance the three-dimensional osteocyte network formation, potentially strengthening the peri-implant bone structure and improving bone-interface resilience [25, 26]. Collectively, implant nanotopography may contribute to peri-implant tissue stability by coordinating mechanotransductive immune calibration, host defense functions, osteoclastogenesis control, and bone-interface reinforcement. This integrated concept is consistent with the proposed role of nanotopography as a multifunctional biointerface that regulates soft-tissue sealing, microbial challenge, innate immunity, and peri-implant bone stability.

## Future perspectives: from cell-instructive nanotopography to active nano-interface regulation

Current strategies for preventing and managing peri-implantitis focus primarily on bacterial biofilm control via mechanical debridement, surface decontamination, and antimicrobial approaches [124]. While these strategies remain essential, peri-implantitis cannot be fully controlled by bacterial eradication alone because disease progression is strongly influenced by soft-tissue sealing, implant-derived foreign body stimuli, innate immune activation, and bone-interface stability. Therefore, future implant and abutment designs should aim to regulate the peri-implant microenvironment in a region-specific manner by coordinating microbial control, soft-tissue integration, immune calibration, and bone-interface resilience. From this perspective, next-generation implant and abutment surfaces should be designed not to optimize a single biological response in isolation, but to coordinate soft-tissue sealing, microbial control, innate immune regulation, and bone-interface resilience within a unified peri-implant homeostatic framework.

Nanoengineered implant and abutment surfaces are promising platforms for this purpose. As discussed above, cell-instructive nanotopographies regulate epithelial and fibroblastic attachment, extracellular matrix organization, macrophage and neutrophil responses, and osteocyte network formation via surface-mediated physical cues and mechanotransduction. Importantly, certain nanotopographies may act beyond simple structural cues by generating active physicochemical stimulation at the implant and abutment biointerface. For example, anisotropically patterned dense titanium nanospikes have been shown to induce electrical cues associated with contact potential differences, resulting in anti-biofouling and mechano-bactericidal effects [19]. This finding suggests that nanoscale architectures can create active physical cues at the biointerface, bridging conventional topographical surface modifications and more actively programmable nano-interface regulation.

**Fig. 3:**
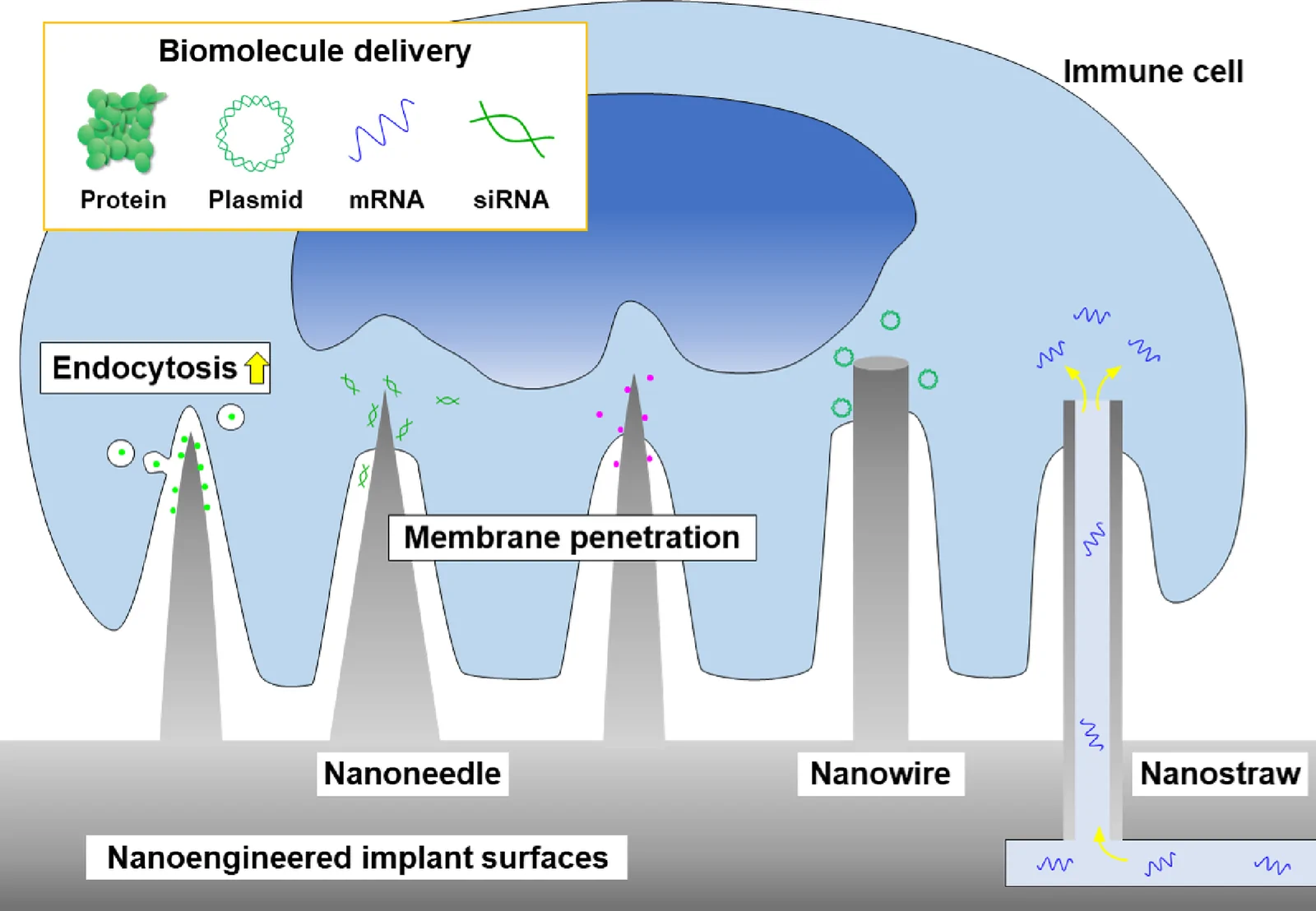
Potential innate immune-cell regulation by nano-enabled biomolecule delivery technologies on dental implant surfaces. Nanoscale delivery platforms, such as nanoneedles, nanowires, and nanostraws enable the delivery of biomolecules including proteins, plasmid DNA, messenger RNA (mRNA), and small interfering RNA (siRNA) through membrane penetration or activation of endocytosis. Although these technologies remain largely experimental in dental implant applications, they may provide future platforms for modulating innate immune-cell functions at the implant–tissue interface

Extending this concept, nano-enabled intracellular biomolecule delivery offers a strategy for directly regulating cellular signaling and gene or protein expression beyond surface-mediated mechanotransduction. However, in dental implant applications, this approach should currently be regarded as a conceptual and experimental extension rather than an established surface technology. Recent advances in surface engineering have enabled the modification of implant surfaces with bioactive molecules such as nucleic acids and proteins to modulate peri-implant inflammation by regulating the behavior of the surrounding cells [125–127]. Additionally, nanoscale delivery platforms, including nanowires, nanoneedles, nanostraws, and nanosyringes, have been shown to deliver DNA, RNA, proteins, siRNA, enzymes, and second messengers directly into the cytosol of various cell types, including immune cells such as macrophages, CD4⁺ T cells, B cells, and T lymphocytes [15, 40–48]. Most of these delivery systems have been developed using non-dental materials or non-implant experimental platforms; therefore, their relevance to dental implantology is primarily mechanistic and translational. These approaches may eventually enable a more precise manipulation of peri-implant immune and stromal cell functions than conventional surface coatings or surface-mediated topographical cues alone (Table 4, Fig. 3).

**Table 4:** Summary of experimental nanoscale intracellular delivery technologies and their conceptual relevance to future dental implant interfaces

Material/delivery platform	Nanoscale feature	Cell type	Delivered cargo/key finding	Conceptual relevance to future dental implant interfaces	Reference
Silicon nanowire-mediated delivery		Fibroblast; hippocampal neurons; neural progenitor cells; HeLa cells	DNA, RNA, and proteins were delivered into the cell cytosol	Demonstrates broad intracellular delivery capability using nanoscale structures	Shalek et al. [40]
		Macrophages CD4^+^ T cells	siRNA was efficiently delivered into the cell cytosol	Provides proof-of-concept for nanoscale modulation of immune-cell gene expression	Shalek et al. [41]
Sharp nanoscale silicon dioxide and titanium-coated tip-mediated delivery		B cells	Plasmids and enzymes were effectively delivered into the cell cytosol through open pores in the cell membranes under irradiation	Suggests possible strategies for intracellular delivery into difficult-to-transfect immune cells	Man et al. [42]
Silicon dioxide nanoneedle array-mediated mechanoporation		T lymphocytes	Large molecules, including GFP plasmids, diffused into the cytosol through transient plasma membrane pores	Supports the concept of direct cytosolic access through transient nanoscale membrane disruption	Dixit et al. [43]
Polycarbonate nanostraw-mediated delivery/electroporation		Ovary cells HEK 293 cells	Plasmids, biomolecules, and Ca^2+^ were delivered into cells; delivery efficiency and dosage could be controlled	Demonstrates nanostraw-based intracellular delivery, dosage control, and the potential to modulate intracellular signaling	Xie et al. [44] Cao et al. [15] Xu et al. [45]
Carbon nanosyringe array-mediated delivery		MCF-7 cells; T47D cells; HepG2 cells; primary lymphocytes; Raji B cells	siRNA, plasmids, fluorescent proteins, and caspase-3 were delivered into hard-to-transfect cells	Provides proof-of-concept for intracellular delivery into multiple difficult-to-transfect cell types, including lymphoid cells	Choi et al. [46]
Porous silicon nanoneedle-mediated delivery		Esophageal adenocarcinoma cells	Nanoparticles diffused throughout the cell cytosol, and biodegradable nanoneedles degraded; injected nanoparticles were detected in a mouse model	Suggests possible relevance of biodegradable nanoscale delivery structures for localized tissue interfaces	Chiappini et al. [47]
Silicon nanowire-associated intracellular uptake		CD4 + T cells; B cells; fibroblasts	Endocytic markers colocalized with delivered biomolecules	Indicates that nanoscale structures may promote intracellular uptake partly through endocytic mechanisms	Chen et al. [48]

**Fig. 4:**
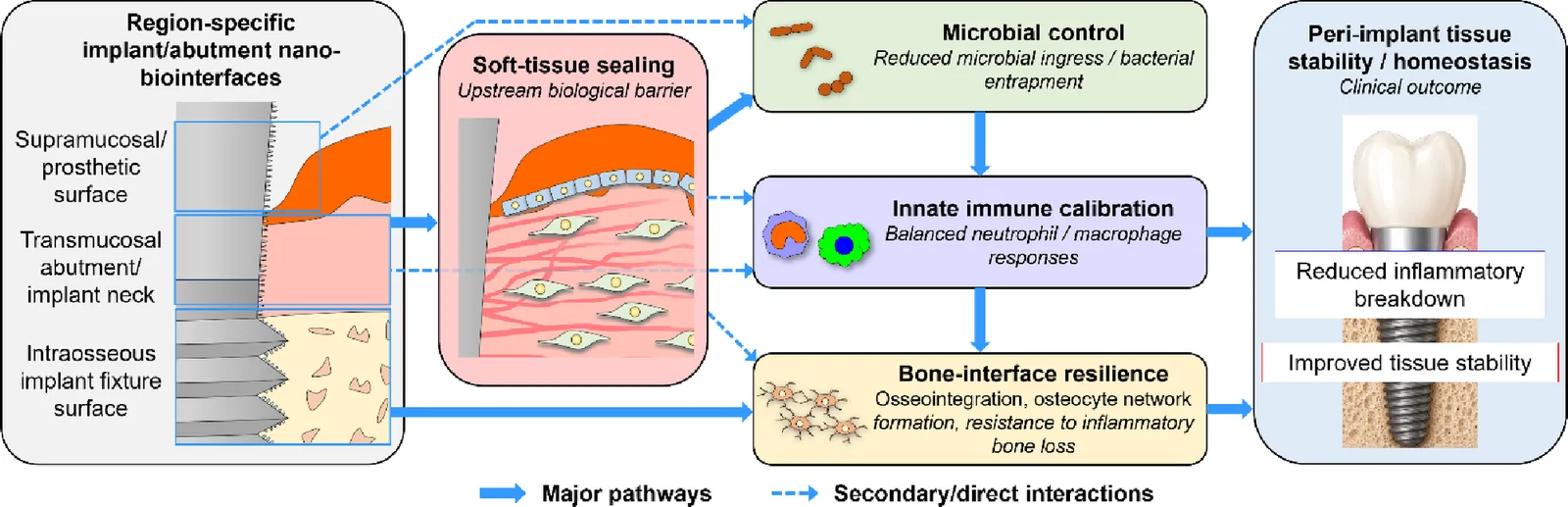
Conceptual framework linking region-specific implant and abutment nano-biointerfaces to peri-implant tissue stability. Nanoengineered implant and abutment surfaces should be interpreted according to their anatomical location and biological exposure. Supramucosal and prosthetic surfaces are primarily associated with cleanability and microbial control; the transmucosal abutment or implant neck region contributes to epithelial attachment, fibroblast adhesion, collagen organization, and soft-tissue sealing; the implant–abutment junction may generate inflammatory stimuli through microgap leakage, micromotion, wear debris, and corrosion products; and the intraosseous implant fixture surface supports osseointegration, osteocyte network formation, and immune–bone coupling. These region-specific biointerfaces interact with soft-tissue sealing, microbial control, innate immune calibration, and bone-interface resilience to determine peri-implant tissue stability and homeostasis. The biological effects of nanotopography should therefore be interpreted as region-dependent and influenced by associated physicochemical surface properties

From a peri-implantitis perspective, such active nano-interface technologies could potentially be used to suppress excessive osteoclastogenic inflammation, enhance macrophage-mediated microbial clearance, promote the resolution of chronic inflammation, and reinforce soft-tissue and bone-interface regeneration. However, most nano-enabled intracellular delivery technologies have been validated in non-dental or non-implant experimental systems, and their direct application in dental implants remains largely conceptual. Translation into clinical implantology will require careful evaluation of the local delivery efficiency, dosage control, long-term surface durability, sterilization compatibility, cytocompatibility, manufacturing reproducibility, and regulatory feasibility, and region-specific placement within supramucosal, transmucosal, implant–abutment junction, or intraosseous environments. In parallel, data-driven materials design approaches, coupled with genome-wide biological analyses of implant integration [128], may help translate molecular-level biological insights into simple, clinically feasible surface-modification strategies [129] for rationally optimized implant surfaces and clinically relevant outcomes (Fig. 4).

Taken together, future implant developments should move toward region-specific nano-biointerfaces that coordinate microbial control, soft tissue integration, immune calibration, and bone-interface stability. Such concepts remain translational, and further preclinical and clinical studies are required to determine whether these nanoengineered strategies can reduce the susceptibility to peri-implant inflammatory tissue breakdown.

## Conclusions

Peri-implantitis is a multifactorial disease driven by bacterial biofilms, disruption of soft-tissue sealing, implant-derived foreign body stimuli, and dysregulated host immune responses. In particular, innate immune cells, such as neutrophils and macrophages, play central roles in shaping the inflammatory and osteolytic microenvironments around dental implants. Current evidence suggests that implant surface nanotopography can influence these processes not only by modulating immune cell mechanotransduction, but also by supporting soft-tissue sealing, reducing microbial retention, and reinforcing bone-interface stability.

Therefore, next-generation dental implants and abutments should not be designed solely according to generalized roughness or coating concepts. Rather, they should be developed as region-specific and functionally active nano-biointerfaces tailored to the biological requirements of supramucosal, transmucosal, implant–abutment junction, and intraosseous regions. In the transmucosal region, such surfaces should promote epithelial and connective tissue sealing while minimizing bacterial entrapment. At the soft tissue immune interface, they should calibrate fibroblast–ECM–macrophage crosstalk and innate immune responses. At the bone interface, they should support osseointegration, osteocyte network formation, and resistance to inflammatory bone resorption. By integrating cell-instructive nanotopography, topography-derived active physicochemical cues, and future experimental biomolecule delivery concepts, nanoengineered implant and abutment surfaces may provide new opportunities to reduce the susceptibility to peri-implant inflammatory tissue breakdown.

## Data Availability

No datasets were generated or analysed during the current study.
